# Institutionalized violence in schools and language displacement: the voices of Mapuche speakers and elders

**DOI:** 10.3389/fpsyg.2024.1485569

**Published:** 2025-01-22

**Authors:** Susan Sanhueza, Fabiola Maldonado, Claudio Díaz, Miguel Friz, Carolina Aroca Toloza, Hector Torres Cuevas

**Affiliations:** ^1^Facultad de Ciencias Sociales, Universidad de Chile, Santiago, Chile; ^2^Facultad de Educación, Universidad de Concepción, Concepción, Chile; ^3^Facultad de Educación y Humanidades, Universidad del Bío Bío, Chillán, Chile

**Keywords:** violence, language, Mapuzugun, Mapuche, culture

## Abstract

This article addresses language displacement as a result of the institutionalized violence experienced by the Mapuche people. The objective is to explore, through the voices of Mapuche speakers and elders, the institutionalized violence encountered in schools and its impact on the displacement of the Mapuzungun language. Through discussion groups, we explored ancestral knowledge, language loss, its intergenerational transmission, and how the teaching of Mapuzungun should be approached for future generations. The results show that the elders possess an immeasurable source of knowledge, being the primary bearers of the culture, and propose an epistemology centered on the unity of language and culture.

## 1 Introduction

### 1.2 Contextual background

The Indigenous population in Chile comprises 2,185,792 individuals, representing 12% of the national population. The Mapuche people account for 84% of the Indigenous population, while the Aymara, Diaguita, Atacameño, and Quechua peoples represent 15%, National Institute of Statistics of Chile, (Instituto Nacional de Estadísticas (INE), Chile, 2017).

There are four significant territorial identities: the Mapuche, which spans the entire territory; the Lafkenche, concentrated mainly in the northern coast; the Williche; and the Pehuenche. This diversity demonstrates that Mapuche identity is not monolithic, and thus, symbolic displacements between territories also occur within the Mapuche identity itself.

According to Nuñez et al. (2022), it is essential to understand territories from an Indigenous perspective, as within the Mapuche Indigenous worldview, the separation between nature and culture is not possible, nor is it between the individual and the community. These are fundamental elements for planning any intercultural program.

In terms of socioeconomic characteristics, the Mapuche people have been confined to poverty and exclusion due to the dispossession of their lands. As stated by Rodríguez et al. (2020), note that the high correlation between Indigenous populations and poverty is a constant in official figures, which many researchers explain as forced poverty, with the State being responsible. As highlighted by Davinson and Candia (2023) provide some data, indicating that 30% of the Mapuche population lives in poverty, 80% of heads of households have less than 4 years of education, and less than 3% of the total population has achieved education beyond secondary school.

One of the most sensitive issues for Indigenous communities is that new generations are not acquiring the language, leading to its gradual extinction. In the case of Mapuche culture, Mapuzungun^1^ is how they relate to the world and construct their identity; through oral communication, they establish both human and spiritual relationships.

Educational institutions have failed to act as a bridge to keep Indigenous languages alive. On the contrary, educational models tend to be monocultural and monolingual, forcing Mapuche children and youth to assimilate the dominant language, resulting in intergenerational language loss.

Indeed, schools promote the marginalization of Indigenous languages in favor of Castilianization, favoring a subtractive bilingualism model. According to Loncón (2020), notes that it is very common for Mapuche students and families to be advised to relegate the use of their “dialect” outside the school environment. This supposed incompatibility between languages and cultures is typical of deeply rooted monolingual conceptions, yet it lacks scientific foundation.

The Chilean State recognizes multiculturalism and multilingualism through various legal instruments: Indigenous Law No. 19.253 of 1993, (Ministerio de Planificación y Cooperación, 1993).; ILO Convention No. 169 on Indigenous and Tribal Peoples in Independent Countries, ratified by the Chilean State in 2008, (Ministerio de Relaciones Exteriores, 2008); the General Education Law of 2009, (Ministerio de Educación, 2009); and Law No. 20.911, which creates the Citizen Formation Plan of 2016, (Ministerio de Educación, 2016).

In the case of the Ministry of Education, the Bilingual Intercultural Education Program (hereinafter BIE) has been implemented in response to a demand from Chilean Indigenous peoples for an education that is culturally relevant and incorporates the languages and cultures of the peoples. However, what could serve as a precursor to practices of recognition and revitalization has instead resulted in various forms of exclusion.

The Mapuche people have been losing their language, especially among children and youth. No studies have explored this issue from the perspectives of Mapuche elders and speakers, who have been responsible for the transmission of the language, which is primarily oral, for many years. Although there are policies and regulations that schools must implement, these legal frameworks have not been successful in reversing intergenerational language loss. Additionally, practices of discrimination and exclusion are still observed against children, youth, and adults from Indigenous communities and/or those who identify as Mapuche.

In this context, the research objectives are: (1) to explore, from the voices of Mapuche speakers and elders, how Mapuzungun is learned and taught to new generations, and (2) to uncover, from their experiences, practices of discrimination and institutionalized violence in schools.

## 2 Conceptual framework

### 2.1 Teaching a second language

The field of second language teaching has evolved through various models over time. In the early 20th century, there was a notable shift toward a mediation-focused approach rather than direct language acquisition, as noted by Ortiz (2020). Initially, this shift promoted additive bilingualism, but it later favored the substitution of indigenous languages. Over time, both strategies were ultimately abandoned. According to Ortiz, this reflects scientific awareness of the diminishing remnants of a language on the verge of extinction, placing greater emphasis on this recognition rather than on any pedagogical objectives that could be fostered within school systems.

This historical context also provides a backdrop for the ongoing loss or displacement of Mapuzugun. Studies that focus on this phenomenon argue that language loss is deeply tied to the colonial dynamics imposed on the Mapuche people, creating fundamental asymmetries between indigenous language speakers and those speaking the dominant language. The colonial legacy, thus, manifests in both linguistic and cultural hierarchies, with indigenous languages positioned at the lower end of these power structures.

In terms of policy, a 2022 study conducted by Chile’s Ministry of Education (Ministerio de Educación de Chile, 2022) highlights that linguistic displacement is closely linked to experiences and narratives of devaluation and discrimination against indigenous languages and cultures. The study identifies two key factors driving this displacement: the role played by educational institutions and the migration of indigenous populations from rural areas to cities. At a global level, the Organización de Naciones UNIDAS (2024), has also recognized that Latin America faces significant challenges regarding the preservation of indigenous languages. The lack of effective policies to support language preservation places Mapuzugun in a precarious position, with only 10% of the Mapuche population still speaking it. Additionally, the generational transmission of the language continues to decline, exacerbating concerns over its survival.

From an educational perspective, the teaching of indigenous languages has been explored through the concept of intercultural communicative competence. This framework, defined by Sumonte et al. (2018), involves a set of cognitive, affective, and attitudinal skills that facilitate communication and interaction between people from different cultural backgrounds. In such intercultural exchanges, the degree to which individuals exhibit these competencies varies, reflecting their proximity or distance from other cultural identities.

A growing body of research advocates for linguistic immersion as a more effective alternative to traditional second language teaching. Immersion programs, as Sumonte et al. (2024), argue, foster environments where the target language is used exclusively, intentionally encouraging communicative interaction. This approach not only accelerates language acquisition but also enables learners to engage more naturally with the language as a living, functional tool.

In parallel, Wang (2023), calls for a decolonizing approach to indigenous language teaching, which foregrounds the value of indigenous knowledge and seeks to integrate these epistemologies into the educational process. One practical example of this is the use of translanguaging, a flexible and dynamic strategy that involves the fluid use of two languages. This approach has gained widespread use in indigenous language teaching programs, particularly in New Zealand, where it empowers learners to draw on both linguistic systems to enhance their understanding and communication in the learning environment.

According to Chew et al. (2023), further emphasize that a decolonizing framework for language teaching is not just a pedagogical tool, but a philosophical stance that acknowledges the identity, intellectual traditions, and cultural agency of indigenous communities. Such an approach aligns with efforts to reclaim and revitalize indigenous languages as part of a broader movement for cultural empowerment.

A critical aspect of successful language education is the role of the teacher or linguistic mediator. As noted by Makeleni et al. (2023), teachers who exhibit higher levels of self-efficacy are more likely to adopt positive attitudes toward both their students and their own teaching. This positive outlook can significantly influence the success of indigenous language education, particularly when teachers are engaged in fostering an inclusive and supportive learning environment.

In conclusion, language teaching encompasses far more than linguistic proficiency; it is deeply intertwined with cultural practices that shape and give meaning to intercultural communication. For the Mapuche people, the teaching and preservation of Mapuzugun are not only linguistic endeavors but also acts of cultural resilience, framed by the historical context of violence and marginalization.

### 2.2 Structural and symbolic violence

The educational system in Chile has long functioned as a mechanism for both cultural assimilation and the perpetuation of social inequalities. On one hand, it systematically excludes, devalues, and stereotypes indigenous knowledge and culture. On the other hand, it restricts social mobility by imposing barriers that prevent Mapuche children and youth from succeeding within the system. This dynamic has resulted in a lower accumulation of educational capital among indigenous communities, exacerbating their marginalization.

A key challenge to the recovery of indigenous languages, such as Mapuzugun, is their visibility and promotion within the educational system. However, the national curriculum in Chile remains rooted in monocultural ideals that fail to address the needs of indigenous populations. As stated by Quintriqueo and Arias (2019), argue that bilingual intercultural education policies have been largely ineffective because they were designed for indigenous students, rather than as a comprehensive solution for all students. Furthermore, the curriculum continues to prioritize a Eurocentric and Western epistemological framework, limiting the inclusion of indigenous knowledge across different levels of education. This exclusion renders indigenous knowledge largely irrelevant for both indigenous and non-indigenous students.

Chile’s national curriculum is standardized across the country, offering little flexibility to adapt to the diverse needs of its student population. This rigidity reinforces legitimized forms of ethnic and cultural discrimination, making schools less inclusive spaces for indigenous children and youth (Rodríguez et al., 2020). The lack of adaptability within the curriculum not only alienates indigenous students but also marginalizes their cultural perspectives and histories.

Poverty further complicates the dynamics within multicultural classrooms. Historically, Chile’s monocultural schooling system was designed to serve the interests of the dominant social classes, often at the expense of the marginalized indigenous population. Quezada (2022), argues that this schooling process was shaped by the need to fulfill the demands of the Western industrial system, with a focus on training children, youth, and adults primarily as future workers. This model of education has contributed to processes of acculturation among indigenous children and youth, leading to the gradual erosion of their cultural identities.

The intersection of poverty, educational rigidity, and cultural discrimination has intensified the acculturation process, resulting in the loss of Mapuzugun and other elements of Mapuche identity. The school system, instead of acting as a bridge to preserve and revitalize indigenous languages and cultures, has often been a force of assimilation, pushing indigenous students to conform to the dominant society’s values and norms.

A thorough review of the literature highlights the importance of focusing future research on the role of language specialists, particularly elders, in the preservation and revitalization of indigenous languages. For the Mapuche people, individuals known as kimches (wise people), machis (shamans), logkos (chiefs), and wewpifes (orators) are key to preserving cultural memory and linguistic knowledge (Quilaqueo and Quintriqueo, 2017). Listening to their voices and incorporating their wisdom into language teaching models is crucial for designing effective programs aimed at preserving Mapuzugun, especially for children and youth.

## 3 Materials and methods

### 3.1 Approach and design

A sociocritical paradigm is chosen (Loza et al., 2020), which is based on social criticism and a self-reflective nature. Researchers acknowledge the power dynamics underlying cultural practices and, consequently, approach the study in that way. In particular, a biographical-narrative research approach is chosen to research about the subjectivity present in the voices of wise and Mapuche speakers. This type of research seemed appropriate due to its retrospective nature centered around the life stories of the participants, who participated in meetings (discussion groups) which had the objective of exploring the social representations of their cultural and linguistic practices. This way, as Bolivar (2012), points out, the social frameworks of interpretation through which people made sense of their experiences were sought. Therefore, more than individual trajectories, the focus was on the value of the history of a group or social phenomenon.

### 3.2 Context and participants

A non-probabilistic intentional sampling method was employed (Cardona, 2002), which involved engaging key informants to facilitate the selection of cases that are both appropriate and rich in information. The samples consisted of either captive informants or volunteers, based primarily on accessibility, ease, and speed of obtaining participant involvement. In total, 25 adults participated in the study, whom we have designated as “Elders and/or Mapuche speakers.” These participants are recognized and esteemed within the indigenous community and serve as cultural bearers through their practices of oral storytelling, which constitute an integral part of the intangible cultural heritage. Of the group, 11 individuals were traditional educators, while 14 were kimches and/or native speakers of mapuzugun. With respect to gender, 16 participants were women.

### 3.3 Data production techniques

For this study, we employed focus groups, a qualitative technique that involves convening a group of individuals to elicit social discourse. While these discussions may take a free format, in our case, they were aimed at identifying, from the voices of the Mapuche elders and speakers, those linguistic revitalization strategies that hold significance for their communities. The questions directed to the participants were focused on the importance of the language, the contextual elements that influence its learning, and the meanings the language holds for Mapuche youth.

According to Criado (2024), a focus group represents a social situation—a group created to construct a common understanding among individuals who do not constitute a pre-existing group; in other words, the participants do not know each other beforehand. The role of the group moderator is solely to induce dialogue, so the interpretive framework emerges from the participants themselves. Four focus groups were conducted, with between 5 and 8 participants, from October to November 2023, as shown in Table 1, with each session lasting approximately 1 h.

**TABLE 1:** Dates and topics of conversation.

Discussion group N^O^	Date	Participants	Topics
1	18/10/2023	8	Ancestral Knowledge Loss of language (inter-generational approach) Language Education
2	25/10/2023	6	
3	06/11/2023	6	
4	07/11/2023	5	

Source: Author’s own elaboration.

### 3.4 Data analysis procedure

The Grounded Theory (GT) method was employed. As Mucchielli (2001) stated, this method aims to inductively produce a theory of a social, psychological, or cultural phenomenon. Moreover, different levels of analysis have been developed: collective readings from the research team, identifying the topics that were relevant to understand how the wise and Mapuche people participated in language teaching, the role they had, and the cultural practices that shaped the transmission of the language.

Then, an inductive coding process was followed, which was based on the theoretical frameworks employed in this study. These were fundamentally about the transmission and teaching of the language and cultural practices. These codes were grouped and commented with memorandums that allowed us to establish relationships between them, team observations, and possible explanations.

At a higher level of complexity, the discourses obtained were contrasted with the theory to reach new interpretations and conclusions.

## 4 Results

### 4.1 The voices of traditional educators: biographies

The majority of the Mapuche speakers and elders were older women whose stories reflect the dispossession of their lands in the southern region of the country, which led them to migrate to the city. They recount having learned the language from their ancestors and recognize the responsibility to pass it on to future generations.

What we have observed is that being born into an indigenous family inherently makes a person a bearer of knowledge and wisdom. For this reason, participants in the focus groups refer to their role as descendants, which obliges them to inherit the identity of their ancestors. This is a territorial identity that connects them to a communal way of life with its own cultural codes. Table 2 presents some of the most illustrative accounts from the discussion groups.

**TABLE 2:** The voices of traditional educators: biographies.

Categories	Declared discourse
Identity Linked to Territory	“I am MM, my origins are from Kowanko, I am a seed curator, and I also give gardening workshops”(GD3).”My paternal grandmother is from the city of Victoria and my maternal great-grandmother has her origins in Chiloé. I studied agronomy, but this year I am teaching Mapudungun in a municipal school in Isla de Maipo.” (GD2)
	“I am from Lumaco, my origins are from Lumaco, I am Nagche, people from the valley. My mother had 4 children, I learned Mapuche from the womb, and I am very grateful to my father for having raised me and spoken to me in Mapudungun.” (GD3)
	“I am MC, I am from Lonquimay, my territory is called Wallen Mapu, my father comes from Peulmapu (Eastern Mapuche territory, Argentina), my mother is from Lonquimay, and we are many siblings, almost 20.” (GD3)
Language as a Family Legacy	“My name is Ll. I am a man from Galvarino, a man from the lowlands (Naqche), but I was born in the city, Santiago. I encountered my Mapuzugun at home, with my maternal grandmother, with my paternal grandfather, and with my maternal grandfather who passed away. They only spoke Mapuzugun, in various places, everywhere. At home, in the city, in the countryside. That’s how I found my Mapuzugun, from an early age. In conversations with the first people, in the ceremonies, in various Mapuche gatherings. Now, I am in the city of Santiago, working on this so that our language does not disappear, so that our language and our way of life do not disappear in the midst of the city.” (GD4)
	“What I learned, I learned from my elders, from my grandparents. Even though both my parents speak it, they don’t speak it at home. I was raised by my paternal grandmother. That’s where what I know and try to learn comes from. Recently, my grandfather passed away, with whom I learned a lot about being Mapuche, with whom I practiced Mapuche traditions, such as traveling and inviting the Machi. Everything I know, I know because of him. So, we feel the loss of the elders. They are a pillar within the family and society as well. We are always learning from them.” (GD1)

Source: Author’s own elaboration.

The data show how the speakers and elders refer to their childhood, family ties, the places they come from, and the meanings these territories hold in their personal lives. In their accounts, the elders and speakers emphasize that language learning has been shaped by family linguistic practices in the context of celebrations, rituals, and forms of social organization.

### 4.2 Cultural and ritual practices

The Mapuche people possess a way of living, being, and thinking that is deeply rooted in community life. For this reason, family conversations are often held in the ruka (Mapuche house). For the interviewees, the ruka allows for the practice of oral traditions and serves as a meeting space to promote their own cultural practices.

Ancestral knowledge is also closely tied to working the land, with workdays beginning very early and prayers being offered to ensure a productive day. The production practices of the Mapuche people are evident in the narratives of the elder men, who emphasize that the time they devote to work is directly linked to meeting their primary needs, as they are self-sufficient and generate their own resources. This contrasts with the stereotype of laziness often attributed to their work ethic^2^. It is important to note that the Mapuche production system is primarily based on rotational crop horticulture, gathering, animal husbandry, and hunting, especially in southern Chile.

Another aspect highlighted in the narratives is the death of a family member, which is always seen as part of a transition process. Those who have passed on are ever-present in their lives and act as mediators between different ontological planes. As such, the departed hold both a sacred and human status. Table 3 illustrates how language transmission occurs in spaces that foster a sense of belonging, such as the *ruka* (traditional Mapuche house) or the communal fire, which brings together Mapuche families as well as their neighbors. The same is true for linguistic practices that accompany daily tasks, productive work, and ceremonial practices, most of which are associated with the supernatural.

**TABLE 3:** Cultural and ritual practices.

Categories	Declared discourse
Linguistic-Cultural Practices in the Ruka	”My mother always taught us to stay very close to our culture. In fact, she created a space within the forest area here in the city, the Naq mapu community, where we painstakingly rescued a Canelo tree by hand. This is where the foye (Canelo) trees are, among many native trees, and in this great city, we have generated a space where we can still maintain our culture. We have our ruka, and in this journey, since I was very young, I have been involved in leadership since I was 20. Now I am 46. I have incorporated the knowledge that my mother passed on to me, and we are constantly strengthening our language, customs, and cultural practices. We do this in our space.” (GD1)
Productive Activities and Interactions	”My father dedicated himself to working the land; working the land is hard because there were no machines for planting or harvesting. For example, the Mapuche used to wake up early, before the sun rose, to receive all the good and to take advantage of good energies for a productive day. At that time, we would perform llellipun; at that time, pillantun, my grandmother said. The machis could be heard in the distance playing their kultrún. In some places, when they play nguillatun, they start at 5 or 7, and people begin to arrive for the ritual.” (GD3) “And also for winter, my aunt had a loom; she made blankets, socks, and vests. I learned about wool there; the sheep’s wool was sheared in November. The wool was stored in sacks. For winter, since it rains a lot in the south, all those memories are with me. For winter, the wool was washed and cleaned, made ready. And there, looking for the wool, it was left to dry, for socks and gloves. The socks my aunt made were sold, and with that, she could buy food.” (GD4)
Death and the Supernatural as Intergenerational Knowledge Transmission	”She went to heaven; it still hurts my heart to think about it. She took us to all these meetings, conversations. Little one, dear one. Now I have dared to come to this meeting. I feel like I make the cross because everything returns to me when she. ‘Come, daughter,’ she used to say, ‘you must learn. There is always something good to learn.’ I was a bit reluctant to come to this meeting because it reminds me too much of her. The rooms, songs, conversations. ‘You came, sister.’ And so it was, up to Playa. The conversation continues; the good thoughts and good things continue in the city. The work will be good, but it should be good like in the past. I refuse to continue being contaminated by everything. And I try to do things as well as possible, even if I make mistakes sometimes. But my mother’s legacy is now a personal commitment of mine.” (GD1)

Source: Author’s own elaboration.

### 4.3 Institutionalized violence: between punishment and discrimination

Violence has manifested in various forms, such as threats against those who speak the language, invisibility within a social group, or punishment aimed at instilling fear or displacing the language and destroying the culture. These practices are frequently reported by participants in discussion groups, who primarily associate them with the use of language in schools or in social spaces beyond their communities.

This situation was particularly acute in confessional educational institutions, especially in schools for poor children run by priests or nuns who sought to evangelize and thereby erase the beliefs of the Mapuche people. Violence operated as a cultural device that facilitated the maintenance of Western culture and the domination of the indigenous people.

Table 4 shows how the language is silenced through physical and psychological punishment, most often when children and young people attend school, as well as the discriminatory practices linked to physical traits, names, and religion. Additionally, there are accounts that illustrate how the language has been “dormant,” yet not entirely lost, and how it can be recovered through practice.

**TABLE 4:** Institutionalized violence between punishment and discrimination.

Categories	Declared discourse
Violence in School and Family	“I lost it when I was 8 years old, with nothing but rulers and sticks. That was the price when I arrived in this city. And as a child, you’re born in one place, but you don’t choose where you’re going to be taken. I don’t blame anyone. The situation just happened, and that’s how it was. But I meet with my siblings, and I don’t lose the essence of recovering my identity as Mapuche” (GD1). “It was my first language; my second language was Spanish. As my sister says here, the nuns taught us with beatings. They were Spanish nuns. So my mother said, my father, who taught me, 1 day taught me, I remember that I started first grade because back then when you were 7 or 8 years old, you started first grade. So, I didn’t know how to read or write because our father taught us about the earth, clay. He would look for a space and that’s where he taught us. My father would look for a stone, ‘just like this,’ and 1 day he taught me, I told him: Father, they punished me, I said. For not reading. And then my father said: Chem niey? I remember there was a picture in the syllabary, it was an eye, so he said, ‘What does this say?’ And I said, ‘Nge’ (eye). My dad hit me because I wasn’t supposed to read it like that. I was supposed to say ‘eye,’ not ‘nge.’ So between my dad and the nuns, they punished me because I entered speaking Mapudungun. I entered speaking Mapudungun at the nun’s school, and the nuns punished me, made me kneel in a corner full of pebbles. ‘You must learn this for tomorrow,’ they would say. So I went to my father to ask him to teach me because my mother didn’t know. She could sign and all that, because they were also taught that they had to speak Spanish, not Mapudungun” (GD1). “Going to school meant great suffering for many boys and girls because the discrimination and mockery toward the Mapuche were severe, and even today it continues. There are many children and even adults who are ashamed of being Mapuche or prefer not to say it. And it’s understandable because the damage caused by the winka was so great that it passed down through generations. For example, there are many chachay and papay (grandfathers and grandmothers) who have scars on their knees because, in the past, they were made to kneel on beans when we spoke our language. Those stories are still very present in our days” (GD2).
Discrimination Practices	“They forbade us from speaking Mapudungun at school, they forbade the boys from playing palín, the girls who wore Mapuche clothing were mocked and discriminated against. So, all of that caused some Mapuche to disconnect from their culture. Then came the issue of evangelization. I don’t speak of God because we Mapuche have the ngen (owners), we don’t have a god. In our territory, we have the owner of the land, the owner of the hill, the owner of the water — everything has an owner, or a female owner. The owner of the water, the owner of the wind, even the fire has an owner. And they have names, each one has a name. So, the whole set of these ngen, living in harmony, creates good living” (GD2).
	“Many of us were forbidden from speaking our language due to the discrimination we could suffer at school, as well as physical punishment. We were prohibited from speaking and continuing to learn Mapuche when the danger and exposure to punishment and humiliation within the school system became greater” (GD3).
Dormant and Silenced Language	“My mom is still young, but even so, at home she doesn’t speak Mapuche. It’s like it’s hard for her to speak. Sometimes she laughs or gets embarrassed, I don’t know, I don’t know what happens” (GD1).
	“On the coast. And there I have my four children, they are all grown up now. My siblings are ashamed to speak Mapuzugun. I speak Mapuzugun. I didn’t want to lose my language, I recovered it, but they forgot it” (GD2).
	“My mom had to learn to speak Spanish to interact with her neighbors. So, she had to speak there and at some point, Mapuzugun was kind of cut off, because in the end, the people she surrounded herself with were winka, not Mapuche people. So clearly, she didn’t lose it, but she stopped practicing it. So, it was like a process, a timeline that we lived, like being more winka ourselves” (GD1).
	“One day I said these words: What the hell am I doing here? And I started to cry. Why did I lose my language? Why am I losing it? One day I wanted to do something, I got up early in that world I was in, in the morning I wanted to do my Llellipun^3^ (traditional Mapuche ceremony). The words didn’t come out. They didn’t come out, my speech was muted. So, I kneeled down, Father Ngünechen (spirit that governs humans, Mapuche God), why? Why me, a Mapuche woman, did I forget Mapuzugun? And there was a flower, I remember. There was a flower. I grabbed it, and I don’t know what I did, I broke the flower. I stayed silent and I said, Father Ngünechen, I’m going to leave. The next day I resigned. I went to my house and told my mother: I forgot Mapuzugun. I forgot my words, I forgot how to speak. Why? And I went to my grandmother. I traveled to Villarrica and told my grandmother: I forgot, I forgot how to speak, I’m speaking in Spanish. My grandmother got angry, she wanted to hit me. I went to a lady who was in Temuco who only spoke to me in Mapuzugun. The old lady said to me: When you spoke Mapuzugun, you were silenced. Why? She said. We are Mapuche. And we speak. And there I recovered my language by speaking, I went to that lady who is no longer with us” (GD1).

Source: Author’s own elaboration.

^3^The Llellipun ceremony is a prayer ritual performed around a canelo tree to ask Ñuke Mapu (Mother Earth) for the well-being of individuals, whether they be patients, workers, family members, friends, or the community in general.

Parents and grandparents also used punishment to prevent children and young people from speaking Mapuzugun, intending to spare them from mistreatment or discrimination at school. Physical force was merely the most visible expression of interethnic violence; however, it was not the only one. The speakers tell us about various forms of caricaturing the Mapuche, such as through their clothing, dances, music, and even children’s games.

According to the speakers, there was an attempt to ensure that indigenous people received the same education as whites and mestizos, giving everyone the same culture. To achieve this, it was necessary not to speak, dress, or feel like a Mapuche. Many eventually accepted these white-imposed definitions promoted by the school, gradually learning a negative, stigmatized, and devalued identity associated with being part of the Mapuche people.

### 4.4 New ways of speaking and being: migration from the countryside to the city

Internal migration is a common phenomenon among all the participants, who originally lived in the southern regions of the country. Mapuche peasant families who managed to keep a piece of their land to produce food attempted to survive in rural areas, often at the cost of sending their children to study or work for wages in the big city.

Table 5 illustrates the processes of internal displacement, mostly driven by land expropriation or the search for better life opportunities, particularly for the younger generation.

**TABLE 5:** Migration from the countryside to the city.

Categories	Declared discourse
Internal Migration	”Due to poverty, many people or families had to migrate to the city, thus creating a generation closely linked to the diaspora of that time. This rural-to-urban migration brought with it various factors that influenced the development and worldview of the Mapuche” (GD3).
	”I was born in the countryside, but as we all know, Mapuche life in the countryside was not always easy. There was a lot of racism, discrimination, and poverty. Being Mapuche was something very bad and for which we suffered a lot, and we still suffer today with the winka” (GD2).
	”Later, when I left the lof (family group), unfortunately, for better or for worse, this system pushes us to study with the winka (non-Mapuche) in the city. I went to Temuco to study, high school, and my life, my way of life, was not the same as the people living in the city. That’s when my language, Mapuzugun, was cut off. The winka wisdom ended Mapuzugun. Later, I went to the eastern side of the territory, to Buenos Aires, to a school, but I never forgot Mapuzugun in my heart and my mind” (GD1).

Source: Author’s own elaboration.

Internal migration arose for various reasons, however, the dispossession of the lands that the Mapuche used for agriculture and self-sustenance was a significant factor. The land gave meaning to the lives of Mapuche families, to the community, and their cultural practices.

## 5 Discussion

As observed, the violence expressed in relationships between different cultural groups within schools is not an isolated incident but rather a form of structural violence. This has led many young people, who today seek to connect with their ancestors, to be perceived as inferior to cultural and political elites. According to Giroux (1985), points out that schools provide various social classes and groups (including Indigenous populations) with the knowledge and skills they need to occupy their respective roles within a labor force stratified by class, race, and gender. Another way this cultural reproduction is expressed is through the distribution and legitimization of forms of knowledge, values, language, and styles that constitute the dominant culture and serve its interests.

The devaluation of Mapuche culture has contributed to the ongoing linguistic displacement through behaviors and prejudices that classify minority languages as inferior or of lesser status. A particular concern is the intergenerational rupture, understood as a fracture or break between young and older generations that hinders the continuity of the practice of Mapuzugun, often due to decontextualized teaching methods or social and historical conditions of domination.

It is pertinent to our study to explain this intergenerational break in language transmission based on research by Mosquera and Carballal (2019), who assert that these processes are reversible if recovery programs with planned and coordinated strategies are implemented. Such programs require the enactment of language policies that facilitate intergenerational engagement, value oral tradition, and promote attentive listening among interlocutors, with Mapuche speakers and elders playing a central role.

Violence is expressed in various forms; for instance, physical punishment is recurrent in the accounts of different speakers. According to Antimil and Olate (2020), “the intergenerational transmission of Mapuzugun has been disrupted due to negative experiences endured by a generation of speakers who faced colonial processes that, among other things, involved schooling that devalued and punished the use of the Mapuche language”.

In our study, we have encountered accounts related to physical and psychological punishment for using Mapuzugun. The frustration stemming from negative experiences and the pressure to assimilate into the dominant culture has created “an epistemological obstacle rooted in both individual and community experiences, which must be overcome in the everyday classroom experience” (Martinuci, 2020).

The results obtained align with the findings of Tapia et al. (2020), who describe the punishments inflicted on Mapuche children in schools. “Punishments were common in schools for speaking Mapuzugun during the 19th century, including whipping aimed at making them forget their customs and ridicule intended to shame them. This reinforced a racist attitude and behavior toward the Mapuche people. In educational contexts, this racism was also expressed through the obligation to learn the official history of dominant groups, the homogenization of territorial and cultural particularities of the Mapuche world, and even the pathologizing of Mapuche Spanish spoken by children as phonological or psychological disorders”.

Identity-related aspects, such as having a Mapuche name or surname, were synonymous with discrimination. According to participants, Mapuche-origin names were associated with moral devaluation, leading many to conceal their origins and attempt to assimilate into Chilean society to avoid discrimination^3^.

Regarding assimilation processes, numerous accounts highlight how schools punished and repressed the use of Mapuzugun, often employing physical violence to achieve the desired effect of denial. Many participants mentioned that it was better not to speak for fear of punishment, and as a result, they gradually forgot their language. Some even felt they were “more” accepted in social life. Assimilation is a strategy of acculturation, implying that Mapuche children or young people prefer to speak Spanish, and by not using their mother tongue, they gradually lose it, which led to the adoption of identity configurations more closely resembling those of the majority group.

The results presented show that speakers never lost their language but that it was “dormant.” Its revitalization emerged when they encountered other speakers, formed a community, and shared common experiences. Engaging in cultural practices began to hold meaning for them.

In the specialized literature, the concept of a “dormant language” has been employed to avoid the controversies surrounding the term “extinct language.” Wittig (2021), uses the term to refer to languages “in which there are no known fluent speakers, as well as cases in which a community has lost its last fluent speakers in recent times.”

Another strategy identified in the accounts of Mapuche elders/speakers was separation, where they preferred to use their own language without showing interest in the one provided by the school. In this way, they avoided making friends or associating with non-Mapuche peers, instead fostering connections with children, young people, and families within the community that reaffirmed their sense of belonging to the group.

One of the most recurrent elements in the accounts explaining the loss of the language is migration to urban areas. Living in urban centers means inhabiting non-traditional spaces where the transmission of the language no longer occurs within a familial context between elders and children, as it traditionally did. The cultural assimilation processes resulting from internal migrations have led to the loss of the language for many participants. Here, we notice that the language is intimately connected to the land, providing identity and a sense of belonging to the collective. We draw on Villalobos (2016), idea that in the Mapuche worldview, “all living beings come from and belong to Mother Earth, which introduces a sense of belonging that contrasts with the basic philosophical concept of modernity, condensed in the idea of autonomy”. This assumption could explain the relationship between language and identity.

As highlighted by Villarroel and Arias (2024), describe this process as intergenerational trauma, where displacement disconnects young people from their worldview and generates a reluctance to identify as Mapuche for fear of exclusion or discrimination. This situation has led many Mapuche to stop transmitting knowledge to their children and grandchildren to prevent them from suffering the same mistreatment they endured.

## 6 Conclusion

In summary, we can conclude that for traditional Mapuche educators and sages the sociocultural, political, and territorial knowledge that should guide the design of Mapuzugun education programs is the Mapuche “worldview”, alluding to the need to reinforce identity and community dimensions of the language, the appreciation of ancestral knowledge, and a sense of territory and spirituality. The biographies of the elderly constitute an immeasurable source of knowledge, being the primary bearers of culture. An important element had to do with the epistemological breakdown in the understanding of language as a “means” to learn culture. On the contrary, the stories of the Mapuche sages and speakers show us an unbreakable relationship for understanding the world. For them, language and culture are a unit.

The school not only forced Mapuche children to silence their language, but they were also made to dress like Chilean people, taking away their traditional clothing and forcing them to wear shoes in a rural context where they preferred to be barefoot to connect with nature. As pointed out by Nahuelpan (2013), points out that these were the first elements that the school and missions tried to tear down. These experiences left marks and shaped current relationships and conflicts.

It is important to mention that the results show us the epistemological break in understanding the language as a “means” to learn culture. In contrast, the accounts of wise and Mapuche speakers show us an unambiguous relationship between language and culture.

On the other hand, the role of the wise and Mapuche speakers (who are mostly elderly people) is necessary for linguistic revitalization, because they are the ones who can teach indigenous knowledge, contextualize, and link traditional contents together with cultural aspects of the natural and social environment in which the family and community have developed.

The results show us that it is necessary to recognize that intercultural education and intercultural didactics have been presented as ways to explain and justify curricular practices centered on cultural reproduction and the reification of knowledge as a means of indoctrination. Therefore, it is not surprising that the participants’ memories are associated with cultural assimilation practices and the denial of their Mapuche identity. As noted by Walsh (2010), states that the only way to counter this vision is to move toward a critical intercultural education that recognizes power dynamics and breaks them through education.

Likewise, the ability to listen, observe, and act is closely related to attitudinal knowledge, and for the Mapuche people, education and the teaching of values are essential. In this sense, an intercultural didactics should contribute to counter racist and discriminatory attitudes by fostering educational and pedagogical principles centered on the sociocultural, political, and spiritual development of the people who participate in educational communities.

### 6.1 Limitations

As limitations, we would like to mention those aspects that have hindered the flow of the research and that could be anticipated in future studies. One of them is that studies on language revitalization are scarce, which resulted in a significant effort at the time of searching for theoretical references that would allow us to contrast the results. The distrust that has developed between the Mapuche people and those who are not also makes it difficult to establish channels of trust that foster genuine communication, which made it necessary to address this aspect before creating the discussion groups.

### 6.2 Projections

Based on the results, we think it is appropriate to propose new research questions that have emerged from the study. For example, we wonder how schools can counteract discriminatory practices associated with the use of the language. We are also curious about what cultural elements should be incorporated into the curriculum of teachers working in multicultural educational centers.

It would be interesting to know what are the most appropriate strategies to evaluate the effectiveness of second language teaching programs. We also wonder how families could continue that educational role at home, especially in language teaching.

From a methodological perspective, it would be interesting to develop longitudinal studies focused on the internal migration processes (associated with indigenous territorial dispossession) that young people have experienced.

## Data Availability

The raw data supporting the conclusions of this article will be made available by the authors, without undue reservation.

## Footnotes

1 The Ministry of Cultures, Arts and Heritage (2019) declares that Mapuzugun is the language of the Mapuche people and is spoken in Chile and Argentina. Upon the arrival of the Spanish, it was spoken from the Choapa River to Chiloé and at its peak it was also a lingua franca. It is an agglutinative language that has 27 phonemes of which 6 are vowels, and it also has variants that are related to the territorial areas in which it is spoken, including pe-wenche, lafkenche, and williche among others.

2 From a Western perspective, the Mapuche are often criticized for working very few hours and are labeled as “lazy.” However, the elders emphasize that they work and harvest to meet their needs, and the idea of living to work is not part of their cultural ethos. They do not gather more than what is necessary.

3 According to data from the Chilean Civil Registry (2004), that year there were around 2,700 Mapuches who re-quested to change their name to a more western one. With this, they sought to feel like another member of society and to be allowed access to greater benefits that, given the discriminatory nature of Chilean society, are very diffi-cult to obtain. For more information see: https://www.elmostrador.cl/noticias/pais/2004/03/15/discriminacion-obliga-a-mapuches-a-optar-por-nombres-huincas/
